# Recurrent posterior shoulder instability—Long‐term results after arthroscopic posterior bone block with capsular reconstruction

**DOI:** 10.1002/jeo2.70166

**Published:** 2025-02-12

**Authors:** Roman F. Karkosch, Juergen Slapar, Tomas Smith, Mathias Wellmann, Marc‐Frederic Pastor, Hauke Horstmann

**Affiliations:** ^1^ Orthopedic Surgery Department Hanover Medical School (MHH) Hannover Germany; ^2^ Orthhoprofis Hannover Germany; ^3^ Department of Orthopedics and Trauma Surgery Staedtisches Klinikum Braunschweig Braunschweig Germany

**Keywords:** arthroscopic posterior bone block, capsular repair, posterior glenoid bone loss, posterior shoulder instability

## Abstract

**Purpose:**

This study reports the long‐term post‐operative clinical outcomes after arthroscopic posterior bone block augmentation with posterior capsular repair.

**Methods:**

Eighteen shoulders (13 patients) with unidirectional posterior shoulder instability were treated with an arthroscopic posterior bone block augmentation and posterior capsular repair in 2011 and 2013 in a single specialized orthopaedic clinic. These patients were invited to participate in a clinical and radiological follow‐up examination to receive long‐term results regarding clinical outcomes, instability, and development of osteoarthritis (OA).

**Results:**

From the initial study group, 13 patients (18 shoulders) could be obtained for a follow‐up examination. The mean follow‐up period was 111 months. At the final follow‐up, two patients (two shoulders) reported recurrent subluxations with a positive apprehension sign. No redislocation was reported. Screw fixation was still in place in seven patients (38.9%). Overall, good clinical outcomes were achieved among Constant–Murley score (77.6 ± 16; *p* = 0.55), Rowe score (67.5 ± 22.1; *p* = 0.34), Walch–Duplay score (58.3 ± 28.2) and Western Ontario Shoulder Index (40.4 ± 23.3%; *p* = 0.96), showing insignificant changes compared with the 2‐year results. Three shoulders developed severe OA (Samilson and Prieto III). No patient required arthroplasty.

**Conclusion:**

Arthroscopic posterior bone block augmentation with posterior capsular repair represents a salvage procedure that can achieve long‐term shoulder stability with overall moderate clinical results. Patients have to be informed about the probable need for implant removal and the high risk of OA development, especially in the presence of pre‐existing cartilage damage, beforehand.

**Level of Evidence:**

Level IV.

**Abbreviations**
CS: Constant–Murley score
CT: computed tomography
MR: magnetic resonance
NRS: Numeric Rating Scale (for pain)
OA: osteoarthritis
PSI: posterior shoulder instability
ROM: Range of motion
RS: Rowe score
SD: standard deviation
SI: shoulder instability
WD: Walch–Duplay score
WOSI: Western Ontario Shoulder Instability

## INTRODUCTION

Recurrent posterior shoulder instability (PSI) is less common than anterior instability but occurs with an increased risk in certain young athletes, such as rowers and climbers with rearward force and leverage [7, 21, 23, 24]. Its treatment remains challenging in spite of various treatment options [22, 23]. The non‐voluntary and voluntary nature of the pathology needs to be distinguished. Therefore, the classification of PSI is difficult as transitions from voluntary to non‐voluntary instability, especially in adolescence, have been reported [15].

Pain and weakness are the leading symptoms, often followed by radiological findings such as labral lesions, posterior glenoid bone loss, and reverse Hill–Sachs defects [18]. Furthermore, a combination of soft tissue and bony lesions is key factors in the development of posterior instability and can be detected with magnetic resonance (MR) arthrography and additional computed tomography (CT) scans [2]. Conservative treatment is effective and has been reported to be successful in over 70% of cases [19].

In cases of structural damage, the primary approach involves strengthening the rotator cuff and managing pain [8]. In the presence of posterior positional functional shoulder instability (SI), electrical muscle stimulation (e.g., a pacemaker device) has shown promising results [19]. In cases of traumatic PSI, posterior capsulolabral repair has been successful [17]. However, it is known from anterior SI that these repairs result in higher rates of recurrent instability and lower union rates in the presence of bony defects [4].

In patients with non‐voluntary instability and failure of soft‐tissue procedures, along with glenoid bone loss, open bone graft augmentation has demonstrated effectiveness in restoring stability [20, 29]. The bone graft extends the glenoid surface and reduces posterior and posteroinferior translation of the humeral head. An additional posterior capsulolabral repair has proven to be beneficial in the presence of inferior instability [28, 29]. Yet, in anterior instabilities, a Latarjet procedure without capsular repair avoids range of motion (ROM) limitations [14].

However, due to its invasiveness and less possible deltoid muscle damage, arthroscopic bone grafting techniques have gained popularity and are now among the commonly used treatment options [3, 5]. Arthroscopic techniques offer better visualization of the bone block position and provide possible treatment options for concomitant pathologies [11, 12].

The aim of this study was to report long‐term data on the outcome of arthroscopic posterior bone block stabilization with concomitant posterior capsular reconstruction for the treatment of PSI. Recurrent instability served as the primary outcome parameter. Secondary outcomes included the development of osteoarthritis (OA) and patient‐reported outcome measures over time. It was hypothesized that recurrent instability would be uncommon with acceptable clinical outcome scores.

## METHODS

The present study was approved by the local ethics committee and is a continuation of a study originally published by Wellmann et al. [29]. All patients from the initial study group who participated in the 2‐year follow‐up (*n* = 24 shoulders in 18 patients) were contacted in advance by phone and provided written informed consent for a long‐term follow‐up examination.

### Study group

Each included patient had received treatment with an arthroscopic posterior bone block procedure using an iliac crest graft and concomitant capsular repair in a single specialized orthopaedic clinic between 2011 and 2014.

Participants underwent a clinical examination by an independent senior orthopaedic specialist who was not the surgeon. The recurrent instability rate was recorded, and outcomes were measured using the Constant–Murley score (CS), the Walch–Duplay score (WDS) and the Rowe score (RS). Furthermore, patients were asked to complete the Western Ontario Shoulder Index (WOSI).

Standard plain x‐rays were analyzed to assess the bone block and screw position and development of OA. The x‐rays were reviewed by two independent shoulder specialists; neither were the operating surgeons.

Regarding the patients' history of instability, one patient was treated directly after an acute traumatic dislocation. The remaining 17 shoulders had a history of chronic posttraumatic SI with more than one dislocation. One patient reported multidirectional instability. All 18 shoulders exhibited a Beighton hyperlaxity score of greater than 6 points and were categorized accordingly. Functional and voluntary SI were defined as exclusion criteria. A single patient (1 shoulder) was treated after a failed soft tissue stabilization, while the remaining 17 shoulders had no history of prior shoulder surgeries. No concomitant pathologies required intervention in the initial operation.

### Surgical technique

All patients underwent an arthroscopic posterior bone block augmentation of the posterior glenoid and a capsular repair, as described in detail by Smith et al. [26]. Briefly, a tricortical bone graft measuring 2.5 × 1 × 1 cm^3^ was harvested from the ipsilateral anterior iliac crest and positioned in alignment with the bone of the posterior glenoid rim, fixed with two 3.5 mm screws in the desired position. Subsequently, the posterior capsule was reinserted to the posterior glenoid rim using suture anchors.

### Statistics

Statistical comparisons were made using SPSS Statistics 24 (SPSS Inc.) and GraphPad Prism Version 9 (GraphPad Software). Descriptive analysis was performed using means and standard deviation. The significance level was set at *p* = 0.05. All reported *p* values are two‐sided and were not adjusted for multiple comparisons.

## RESULTS

### General findings

From the initial study group, 13 patients (18 shoulders, 6 males and 7 females) were available for a final follow‐up examination (75%, *n* = 13). Each patient provided written informed consent. At the time of the examination, the mean follow‐up was 111 months (range: 98–128 months).

Patient demographics are displayed in Table 1. The male participants were significantly older than the females (*p* = 0.0061), with a range from 24 to 56 years at the final follow‐up (see Table 1 for details).

**Table 1:** Population demographics.

Variable	Global population	Male	Female
Sex, *n* shoulders (%)	18	7 (38.9)	11 (61.1)
Bilateral	5	1	4
Mean age at surgery, years (SD)	23.6 (9.2)	30.4 (10.2)	19.3 (5)
Mean age at follow‐up (SD)	32.8 (8.9)	39.6 (10.1)	28.5 (4.7)
Mean follow‐up, months (SD)	111.3 (8.7)	112 (8.9)	101.8 (31)
Implant removal	11	4	7
Return to occupation	18	7	11
Return to sports	10	5	5
Overhead activities	14	5	9

Abbreviation: SD, standard deviation.

All patients were able to continue or start a working career (100%). Seven patients (56%) reported that they were able to return to sports activities, including throwing sports, rowing, climbing, and weightlifting. 61.5% (*n* = 8) stated they would have the surgery again and claimed that they were happy with the outcome.

### Recurrent instability

At a mean follow‐up of 111 months, two patients (two shoulders) reported recurrent subluxations with a positive apprehension sign. No complete dislocation was reported during the follow‐up period.

### Radiographic results

Graft non‐unions were not observed. Hardware removal had been performed in 11 patients due to posterior shoulder pain (61.1%). OA was classified according to Samilson and Prieto's radiological classification in the standard anteroposterior view x‐ray. At the time of surgery, only two shoulders (both male, aged 32 and 28 years) exhibited Grade 1 OA. At the final follow‐up, this number increased to 10 (55.6%) with some degree of OA. Table 2 provides an overview of the development of OA:

**Table 2:** Degree of osteoarthritis (OA; Samilson and Prieto).

Degree of OA	None	Mild	Moderate	Severe
*n* =	8 (16)^a^	5 (2)^a^	2 (0)^a^	3 (0)^a^
%	44.40%	27.80%	11.10%	16.70%

^a^ Indicates the degree of OA at inclusion.

**Figure 1:**
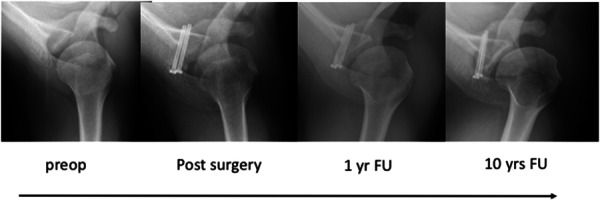
Standard axial radiography over time in a patient without screw removal. FU, follow‐up.

The documented range of OA development was large and depended on the preoperative conditions as well as exposure to external factors such as sports and work. The number of dislocations prior to the surgery and its influence on the degree of OA could not be recorded. However, most shoulders revealed no signs of OA on plain radiography, while older patients tended to show severe OA. Figure 1 presents an example of a patient's axial radiographs over time.

Bone block resorption was difficult to assess, especially in cases with screw removal during the interval. In 13 cases, a bone block was still visible in the axial view. Five cases (27.7%) were suspected to have bone block resorption. In these cases, a CT scan would have been desirable but was not part of the study protocol due to concerns over radiation exposure.

### Functional outcomes

**Figure 2:**
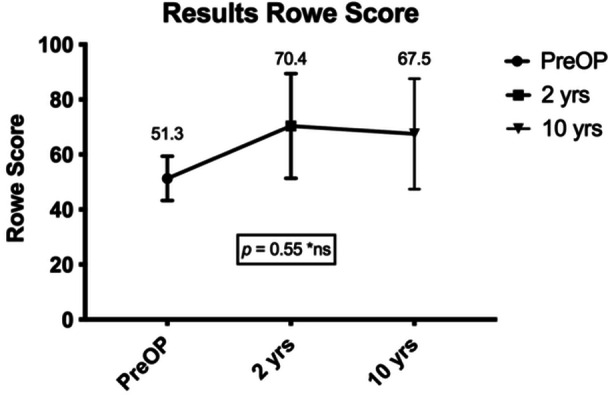
Display of Rowe score over time.

**Figure 3:**
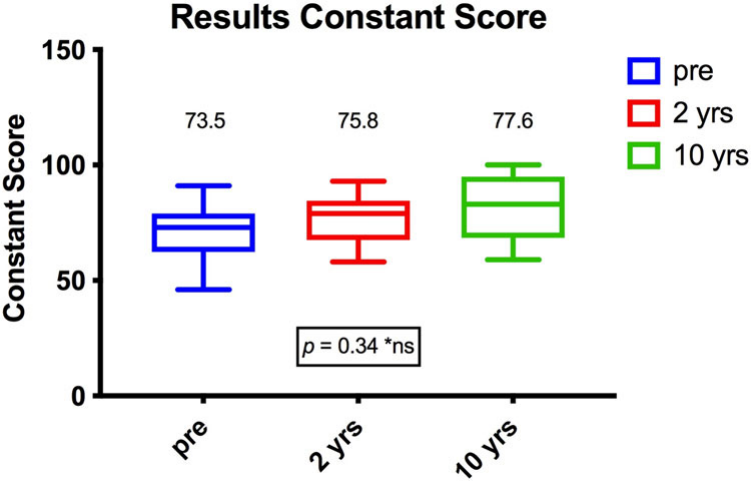
Display of Constant–Murley score over time.

All patients were examined with the use of common shoulder assessment tools and were compared to the results they had achieved 2 years after the surgery. The RS slightly decreased over this period of time from 70.4 to 67.5 points (*p* = 0.5464) (see Figure 2). The CS showed an insignificant increase of 1.8 points to 77.6 (*p* = 0.3396) at the final follow‐up (see Figure 3). Finally, the WOSI score changed insignificantly from 35.7% to 40.4% (*p* = 0.9565). When compared to the preoperative outcome measures, significantly better results were achieved for the RS (*p* = 0.0115) and WOSI (*p* = 0.0025). Table 3 provides an overview of all evaluated outcome measures.

**Table 3:** Display of outcome measures.

Outcome measures^a^				
	Preoperative	2 Years	10 Years	*p*
Constant score	73.5 (13.3)	75.8 (12.3)	77.8 (15.6)	0.34
Rowe score	51.3 (8)	70.4 (19)	67.5 (22)	0.55
Western Ontario Shoulder Instability score	63.1	35.7	40.4	0.96
Walch–Duplay score	‐	‐	58.3 (28.1)	‐

Abbreviation: SD, standard deviation.

^a^ Values are presented as mean ± SD unless otherwise indicated. Walch–Duplay scores were not recorded before surgery and at the 2‐year follow‐up. *p* Values are calculated based on the 2‐year results.

### Re‐operation and complications

Revision surgeries for instability were not performed. At the final follow‐up, seven shoulders still had the screws in place, while screw removal due to local soft tissue irritation was performed in 11 shoulders during the follow‐up period. One patient required a combined procedure with arthrolysis due to stiffness and loss of ROM.

### Pain

Pain at rest was reported in eight cases, with an average Numeric Rating Scale (NRS) score of 4. Thirty percent of patients (*n* = 5) reported frequent use of non‐steroidal anti‐inflammatory drugs, and two patients required tricyclic antidepressants for pain management. Pain during motion and under load‐bearing conditions was present in over 60% of cases (11 patients).

## DISCUSSION

The principal finding of this study is that arthroscopic posterior bone block augmentation with posterior capsular repair can achieve long‐term shoulder stability with moderate clinical results and should be considered a salvage procedure due to the high risk of OA development, even in young patients. It represents a tool in the challenging treatment of non‐voluntary PSI when non‐operative treatment and soft‐tissue procedures fail.

Recurrent instability is naturally the primary outcome parameter in the treatment of any SI. The study demonstrated that this is not a significant concern in the long term, as only two patients reported subluxation and positive apprehension signs. Open posterior bone block procedures, as described by Villefort et al., showed a higher rate of recurrent instability of 31% in their cohort [27]. Mojica et al. found a re‐dislocation rate of 4.4% and nearly 10% for recurrent instability in their systematic review of open and arthroscopic posterior bone blocks [18]. At the 2‐year follow‐up, one patient experienced a recurrent dislocation, while two patients exhibited a posterior subluxation with positive posterior apprehension. This recurrence rate of 12.5% was comparable to the literature [29].

The findings of the present study showed a stable development of functional outcome parameters comparable to those at 2‐year post‐surgery [29]. In contrast to previous studies for recurrent instability, revision surgeries apart from hardware removal were not an issue in this cohort [10]. However, hardware removal was performed frequently, and patients need to be aware of the high probability of a planned revision surgery. Hardware‐related complications, such as shoulder pain, OA and humeral head impingement, are well‐known in bone grafting for anterior shoulder stabilization with cannulated screws [1, 16]. Therefore, Boileau et al. suggested using suture‐button fixation [6]. Additionally, in 2021, Hachem et al. introduced an all‐arthroscopic Latarjet technique using high‐strength sutures, which generated good stability [13]. Given the high rate of hardware‐related re‐operations in the study group, the use of a screw‐free fixation method is desirable for posterior shoulder stabilization as well. Graft resorption, exposing the screws in arthroscopically assisted posterior bone block augmentation, is common [9]. Therefore, CT scans would have been desirable, to evaluate the condition of the bone graft in the long term. Due to ethical concerns related to radiation exposure, CT scans were not included in the study protocol for this young study cohort. Plain radiographs could not reliably illustrate graft resorption in our examinations.

As shown in previous studies, the development of OA is a significant issue in the surgical treatment of posterior instability using bone block techniques [3, 9, 25]. The long‐term results of the present study demonstrate a high prevalence of OA accordingly. Approximately every second patient developed some degree of OA. However, no shoulder arthroplasty was performed during the study period. Villefort et al. had to exclude two of their participants with open bone block procedures due to the need for arthroplasty. Yet, the progression of arthritis in 55.6% of the patients in our cohort was comparable to the progression rate reported by Camenzind et al. [9]. In their evaluation of posterior bone blocks using CT and a follow‐up of at least 2 years, arthritis progression was observed in 47% of the patients. In patients with no preoperative signs of OA, only three shoulders showed a Grade I OA according to Samilson and Pietro at 2‐year follow‐up [29]. At the same time, a significant percentage of patients (44% of the cohort) did not develop any radiological signs of degenerative changes in the long term. It remains unclear why the development of OA is so widespread and difficult to predict. The influence of bone block positioning on the development of OA in this examination remains debatable, as post‐operative CT scans were not available. Routine CT scans post‐operatively should be considered in future cases. The original study exhibited radiographic remodelling in all 18 shoulders of the follow‐up group 2 years after surgery. With the knowledge of high OA rates, pre‐existing cartilage damage and concomitant pathologies should be highlighted when posterior bone block stabilization is considered.

It would be interesting to determine whether patients who experienced more dislocations preoperatively also developed OA more frequently over the years. Unfortunately, we were not able to include this information in the study, as it was not recorded preoperatively.

Finally, post‐operative pain is a key factor in the patient's perception of a successful surgery. Pain after PSI is not uncommon, as Mojica et al. found 11.6% of patients with residual pain in their review. They did not classify pain further according to the numeric or visual analogue scale. In the present study, pain among all participants was generally low (NRS 2.3) but occurred at rest in eight cases (44.4%).

## LIMITATIONS

This study has certain limitations. Due to the low incidence of the need for posterior bone block procedures, it was difficult to create a large study population. Despite all efforts to contact the entire original study group, only 75% (*n* = 18 shoulders) of the patients were reached. The pathology of PSI is still subject to debate, and the distinction between voluntary and non‐voluntary instability is not always clear, leading to a heterogeneous patient group.

Furthermore, the cohort did not have a control group and the age differences among the participants at the time of surgery ranged from 15 to 40 years, with an outlier in the male study population. The lack of CT scans made an evaluation of the actual bone condition at the final follow‐up impossible, but it would be of great interest. The degree of glenoidal bone loss at the time of surgery was not taken into consideration. Finally, due to the long follow‐up period, increasing age may have influenced the outcome itself.

## CONCLUSION

Arthroscopic posterior bone block augmentation with posterior capsular repair represents a salvage procedure that can achieve long‐term shoulder stability with overall moderate clinical results. Patients have to be informed about the probable need for implant removal and the high risk of OA development, especially in the presence of pre‐existing cartilage damage, beforehand.

## AUTHOR CONTRIBUTIONS

All authors contributed to the study conception and design. Material preparation, data collection and analysis were performed by Roman F. Karkosch, Marc‐Frederic Pastor and Hauke Horstmann. Roman F. Karkosch and Juergen Slapar examined the patients. Tomas Smith and Mathias Wellmann helped supervise the project. The first draft as well as the revisions of the manuscript were written by all authors. All authors read and approved this final manuscript.

## CONFLICT OF INTEREST STATEMENT

The authors declare no conflicts of interest.

## ETHICS STATEMENT

The approval was obtained from the local ethics committee of Hanover Medical School. The procedures used in this study adhere to the tenets of the Declaration of Helsinki (9743 BO S 2021). Informed consent was obtained from all individual participants included in the study.

## Data Availability

The data that support the findings of this study are not openly available due to reasons of sensitivity and are available from the corresponding author upon reasonable request. Data are located in controlled access data storage at Hanover Medical School, Germany.
